# Matrine-loaded Nano-liposome Induces Apoptosis in Human Esophageal-squamous Carcinoma KYSE-150 Cells

**DOI:** 10.2174/0113816128306477240625101849

**Published:** 2024-07-11

**Authors:** Kai Zhao, Yun Cai, Faisal Raza, Hajra Zafar, Liang Pan, Xifeng Zheng, Wenjie Xu, Ran Li, Feng Shi, Yongbin Ma

**Affiliations:** 1 Department of Gastroenterology, Jintan Hospital Affiliated to Jiangsu University, Jintan 213200, P.R. China;; 2 School of Pharmacy, Shanghai Jiao Tong University, Shanghai 200240, China;; 3 School of Pharmacy, Jiangsu University, Zhenjiang 212013, P.R. China;; 4 Department of Central Laboratory, Jintan Hospital Affiliated to Jiangsu University, Jintan 213200, P.R. China

**Keywords:** Matrine, nano-liposome, esophageal-squamous, apoptosis, KYSE-150, nanoliposome

## Abstract

**Introduction:**

Esophageal-squamous Cell Carcinoma (ESCC) is often diagnosed at the middle or late stage, thus requiring more effective therapeutic strategies. Pharmacologically, the anti-tumor activity of the principal active constituent of *Sophora flavescens*, matrine (MA), has been explored widely. Notwithstanding, it is significant to nanotechnologically enhance the anti-tumor activity of MA in view of its potential to distribute non-tumor cells.

**Methods:**

Herein, MA-loaded Nano-Liposomes (MNLs) were prepared to enhance the effect of anti-ESCC. The MNL showed a smaller sized particle (25.95 ± 1.02 nm) with a low polydispersed index (PDI = 0.130 ± 0.054), uniform spherical morphology, good solution stability, and encapsulated efficiency (65.55% ± 2.47). Furthermore, we determined the characteristics of KYSE-150 cells by cell viability assay, IC_50_, Mitochondrial Membrane Potential (MMP), Western blot, and apoptotic analysis, which indicated that MNLs down-regulated the cell viability and IC_50_ in a concentration-dependent manner and induced a significant change in JC-1 fluorescence from red to green.

**Results:**

The above observations resulted in increased Bax and Caspase-3 levels, coupled with a substantial decrease in Bcl-2 and apoptotic promotion at the advanced stage compared with MA.

**Conclusion:**

Based on these results, MNLs may serve as a more effective and promising therapeutic option for ESCC.

## 1 INTRODUCTION

Of all the malignancies of gastro-intestines, Esophageal Cancer (EC) is considered the most aggressive tumor. With regard to the causes of tumor-associated mortality in men, EC is ranked the highest worldwide [1]. The cancer of the esophagus is subdivided into Esophageal-squamous Cell Carcinoma (ESCC) and Esophageal Adenocarcinoma (ECA) based on clinical histopathology. In China, roughly 90% of all yearly cases of EC are of ESCC [2]. Available literature points to complex risk factors of ESCC, wherein the suspected and confirmed factors are smoking tobacco and betel quid, increased abuse of alcoholic beverages, low status of socioeconomic, fatty diets, eating of contaminated fruits and vegetables, Polynuclear-Aromatic Hydrocarbons (PAHs), pickled vegetables, higher body mass index, frequently taking hot beverages and foods, poor oral health, lack of micronutrients, and genetic and reproductive-related hormonal factors [3]. Of note, ESCC is diagnosed during the middle or late stage in most patients; hence, it results in poor prognosis. Current treatment options for ESCC are immunotherapy, neoadjuvant chemotherapy and radiotherapy, targeted therapy, and esophagectomy. Chemotherapy and radiotherapy are the main treatments for ESCC. However, after prolonged chemotherapy or radiotherapy, it will develop resistance to drugs, which results in serious side effects. Moreover, it has been reported that although platinum-based chemotherapy inhibits ESCC cell growth, cisplatin and oxaliplatin, as the most effective agents in the chemotherapy, cause serious side effects, including nausea, vomiting, diarrhea, and suppression of hematopoiesis. In spite of continuous advancement in treatment methods, the 5-year survival rate of ESCC is still roughly 30-40% [4]. Based on this notion, a novel approach to developing more effective treatment options for ESCC is urgently needed.

**Fig. (1):**
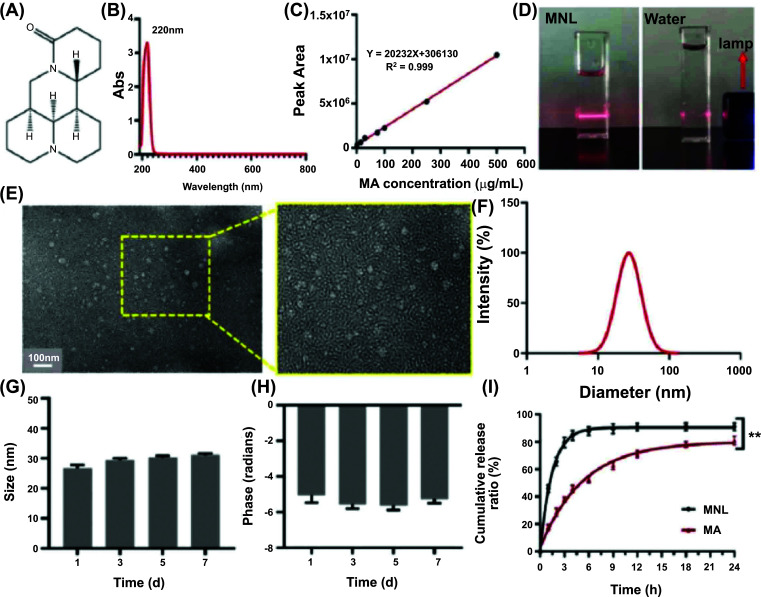
The preparation and characterization of matrine (MA)-loaded nano-liposomes (MNLs). (**A**) The chemical structure of MA featured a tetracyclic quinolizidine alkaloid framework. (**B**) UV spectroscopy of MA showing a maximum absorption peak at 220 nm. (**C**) Standard curve of MA with the equation Y = 20232X + 306130 and R^2^ = 0.999. (**D**) MNL suspensions appeared as the homogeneously colorless solution, forming a straight beam without visible water boundaries. (**E**) Transmission Electron Microscopy (TEM) image showing MNLs as spherical droplets with particle sizes around 30 nm. (**F**) Dynamic light scattering (DLS) analysis of MNLss. (**G**, **H**) Particle size and Z-potential of MNLs showed no significant changes from day 1 to day 7. (**I**) The cumulative release of MA from the liposomal formulation in PBS (pH = 7.4) was significantly higher (**P < 0.01, compared with MA) than that of unencapsulated MA.

In view of its excellent beneficial effects and minimal toxicity, Traditional Chinese Medicine (TCM) has attracted much attention from scientists worldwide [5]. As an example of TCM, *Sophora flavescens* contains matrine (MA, C_15_H_24_N_2_O, the chemical structure is shown in Fig. **1A**) as the principal active constituent, wherein it is grouped under alkaloids with various pharmacological benefits. Previous studies have demonstrated its various benefits, including anti-oxidant [6], anti-inflammation [7], anti-fibrotic [8], and anti-tumor activities [9]. In addition, MA was reported to induce suppression of growth and apoptosis of different tumor cells, including glioma [10], nasopharyngeal carcinoma [11], multiple myeloma [12], hepatoma [13], cervical cancer [14], melanoma [15], colon cancer [16], oral squamous cell carcinoma [17], lymphoma [18], breast cancer [19], and gastric cancer cells [20]. Meanwhile, the apoptotic effect of MA against human ESCC has been well-documented [21]. It was found that MA inhibits cell proliferation and leads to significant apoptosis *via* the ROS-mediated mitochondrial intrinsic apoptotic pathway. Moreover, MA causes KYSE-150 cell death characterized by alterations in the F-actin cytoskeleton, cell morphology, and surface ultrastructure. However, the anti-tumor effect of MA is possibly diminished because of the potential of the alkaloid to distribute to non-cancerous tissues *via* crossing the blood vessels. Tumors have blood vessels with a pore size of hundreds of nanometers, which is higher than the capillaries of healthy tissue. The lipidation of the drug allows particles of 10-100 nm to pass through and accumulate in the tumor tissue while minimizing their toxicity in healthy tissue. In order to enhance the anti-tumor activity and increase bioavailability of MA in ESCC, we prepared MA-Loaded Nano-Liposomes (MNLs). Available literature has reported increased delivery and accumulation of drugs in tumors by nano-liposomes *via* underlying mechanisms like enhanced permeability and retention (EPR) [22, 23].

Thus, we prepared MNLs using the membrane-sonic method and characterized them. Subsequently, we evaluated the anti-tumor activity of MNLs in ESCC with human ESC KYSE-150 cells as an *in vitro* model, with emphasis on their apoptotic impact. The findings of this work may provide a more effective and promising approach for treating ESCC.

## 2 MATERIALS AND METHODS

### 2.1 Analytical Analysis of MA Concentration with High-performance Liquid Chromatography (HPLC) Technique

Analytical analysis of MA was carried out using the appropriate technique, wherein we developed an HPLC method. The HPLC instrument was composed of a symmetrical C_18_ column (with dimensions of 5 μm and 4.6×250 mm, manufactured by Waters, USA). We carried out the analysis under the chromatographic conditions, namely the volume of sample injection (20 μL) with the solution of methanol and water as mobile phase (9/1, vol/vol), which flowed at a rate of 0.8 mL/min. Other factors considered were wavelength of detection (220 nm) and column temperature (25℃).

### 2.2 Procedure for MNL Preparation

The procedure for the preparation of MNLs is as follows: we dissolved MA (20 mg) and soybean lecithin (0.6 g) in 10 ml of CHCl_3_ and conducted ultrasound (Kunshan Ultrasonic Instruments Co., Ltd) at 35℃ for 10 min. The above solution continued to be sonicated until clarification, after which cholesterol (0.1 g) was added. After shaking, most of CHCl_3_ was removed by rotary evaporation (Heidolph Co., Germany) at 45℃ and 100 rpm for 30 min to form a lipid film. Later on, we added the appropriate amount of deionized water to top up the total volume to 10 mL before shaking it to obtain the uniform solution, which was further ultrasonicated for 5 min. The prepared MNLs were stored at 4℃ for *in vitro* property evaluation.

### 2.3 Characterization of MNL

#### 2.3.1 Determination of Nano-liposomal Size and Z-potential

After the ultrasonication process, we diluted MNLs (2 mg/mL) 10 times. Afterward, we accordingly measured the nano-liposomal size, polydispersed index (PDI), and Z-potential of MNLs with a laser analyzer for particle size (NanoBrook 90 plus, Brookhaven Instruments, USA). Every measurement was taken three times.

#### 2.3.2 Morphological Observation with Transmission Electron Microscopic (TEM) Technique

Morphological observation of nano-liposomes was accomplished with TEM (JEM-2100, Japan). For TEM analysis, diluted MNLs were added onto a copper grid, followed by negative staining of phosphotungstic acid and drying with an infrared lamp. Observation of morphology and pictures were carried out after placement of the copper grid with MNLs into the TEM machine.

#### 2.3.3 Estimation of Drug Loading and Encapsulated Efficiency of MNL

The MA-loaded nano-liposomes were diluted with 10% TritonX-100 to release encapsulated MA from liposomes prior to the estimation of drug loading (DL%) and encapsulated efficiency (EE%). In addition, an equal amount of MNL solution was placed into an ultrafiltration tube (MWCO = 30 kDa) before 30 min of centrifugation at 5000 rpm. Free MA in the outer tube was assumed to be not encapsulated into liposomes. Therefore, the quantity of MA in the liposomes was regarded as the total quantity of MA after the disruption of liposomal vesicles minus the quantity of free MA. Analysis of MA content was accomplished using the HPLC method. The EE% and DL% were calculated as follows:

EE% = (the quantity of MA in nano-liposomes)/(the quantity of MA loaded initially) × 100%; DL% = (the quantity of MA in nano-liposomes)/(the quantity of MA loaded into nano-liposomes) × 100%.

### 2.4 *In vitro* Release of MA from MNL

*In vitro* release analysis was conducted in a dissolution medium, such as the solution of phosphate buffer (PBS, pH 7.4). Later on, 2 mL of MA and MNL were suspended in a dialysis membrane bag (MWCO =30 kDa) before the bag was sunk, and MA was released into 50 mL of PBS solution under 48 h of agitation (100 rpm). Afterward, we collected the dissolution medium (2 mL) at the indicated interval, while a fresh medium was replenished at the same volume. HPLC was utilized to analyze MA in the collected medium. The cumulative release (CR%) of MA loaded into nano-liposomes was calculated as a ratio to the total MA with the following equations: 

CR% = (cumulative amount of MA in the release medium)/(total amount of MA in nano-liposomes) × 100%.

### 2.5 Culturing of KYSE-150 Cells

The Yubo Biotech. Co. (Shanghai, China) supplied the human ESCC KYSE-150 cells. Under the humidified atmospheric condition with 5% CO_2_, we carried out culturing of the cells at 37℃, with the medium being RPMI-1640, wherein it contained fetal-bovine serum (FBS, 10%).

### 2.6 Viability Assay

To evaluate the viability of MA and MNL towards cells, we seeded the KYSE-150 cells onto 96 well plates before 24 h of culturing. Later on, the cultured medium was accordingly replaced with the FBS-free medium comprising of MA and MNL at varied concentrations of MA (1, 5, 10, 25, 50, and 100 μg/mL) for another 24, 48, and 72 h. The control for this experiment was cells that were incubated with FBS-, MA-, and MNL-free medium. At the specified time, we placed a solution (20 μL) of 3-(4,5-dimethyl-2-thiazolyl)-2,5-diphenyl-tetrazolium bromide (MTT, 0.5%) onto the plates. Incubation was accordingly carried out for 4 h after the addition of MTT, and 10 min after, we added DMSO (100 μl). An automatic microplate reader (Bio-Tek, USA) was employed to measure absorbance at 490 nm. The following equation was applied to calculate the viability of cells: 

Cell viability (%) = (OD_sample_/OD_control_) × 100%.

### 2.7 Imaging and Analysis of Mitochondrial Membrane Potential (MMP)

Incubation of KYSE-150 cells treated with MA and MNL was carried out for 30 min with a JC-1 dye for MMP. Meanwhile, KYSE-150 cells without any treatment were stained with JC-1 dye as blank control. After fixation, the cells were imaged under a fluorescence microscope (Nikon, Japan). To quantitatively record the change in the MMP of the cells, we performed 30 min of incubation at 37℃ with rhodamine-123 (5 μg/mL) before the fluorescence signal was analyzed *via* Image J.

### 2.8 Apoptotic Rate and Flow Cytometric Analysis

Briefly, we incubated the KYSE-150 cells for 48 h with MA and MNL (25 μg/mL). Meanwhile, KYSE-150 cells without any treatment were incubated for 48 h as blank control. Subsequently, we incubated KYSE-150 cells with PBS comprising of propidium iodide (PI, 0.25 μg/mL) after the above treatment. Using a flow cytometer, we quantified the viable cells (PI-negative) *via* the flow cytometric technique. Later on, we used annexin V-FITC to stain (lasted for 15 min) the treated cells in annexin V staining buffer to evaluate apoptosis at ambient temperature before counterstaining with PI. Afterward, we performed flow cytometry to determine the apoptotic rate, wherein the analysis of profiles was accomplished with FlowJo software.

### 2.9 Western Blot

A lysis buffer of radio-immunoprecipitation assay (RIPA) was employed for the lysis of MA-, MNL-treated cells, and untreated cells. Based on the instruction of the manufacturer, we appropriately quantified the concentrations of protein with a BCA kit. Later on, we loaded protein (4 μL) in each gel lane of sodium-dodecyl sulfate-poly-acrylamide-gel electrophoresis (SDS-PAGE, 10%) prior to separation of the sample. Subsequently, we transferred the proteins to membranes of poly-vinyl-idene di-fluoride (PVDF). To obtain the perfect capability of the antibodies to bind antigens, we carried out 1 h of blocking of membranes at ambient temperature with bovine serum albumin (BSA, 5%). Later, all-night incubation of the blots with primary antibodies, namely Bax, Bcl-2, and caspase-3 (supplied by cell-signaling technology, USA; 1:1000), was accomplished with the control being β-actin. Membranes were visualized and photographed by the gel imager.

### 2.10 Statistical Analysis

All analyses were performed using GraphPad Prism software (version 8.0). Expression of experimental results was achieved with mean and standard error of the mean (SEM). Multiple groups were compared using a one-way analysis of variance (ANOVA). In *in-vitro* release profiles, the data of cumulative release was analyzed using two-way ANOVA with time as a repeated measures factor and free-MA or MNL as a between-subject factor. In the cell viability assay, the cell viability was analyzed using two-way ANOVA with drug concentration as a measure factor and free-MA or MNL as a between-subject factor. Statistical analysis was performed by applying Student’s t-tests to compare the mean between the two groups. We defined a significant level of acceptance to be *P* < 0.05.

## 3 RESULTS

### 3.1 Preparation and Characterization of MNLs

Nano-sized drug carriers help to enhance the absorption of drugs and improve their therapeutic effects. To enhance the anti-tumor effect of MA, we prepared MNLs as nano-liposomes were reported to increase the delivery and accumulation of drugs in tumors *via* underlying mechanisms like EPR [22]. The results of UV spectroscopy scanning showed that the peak absorption of MA was 220 nm (Fig. **1B**). Thus, the HPLC method for analysis of MA was established, while the standard curve of MA was Y=20232X+306130 (R^2^ = 0.999) (Fig. **1C**). The results of intra- day/inter-day precision, recovery test, stability, and repeatability are shown in Tables **1**-**5**. Afterward, MNLs were successfully prepared in small sizes and uniform circular shapes. As shown in Fig. (**1D**), MNL suspensions appeared as homogeneously colorless solutions and formed a straight beam without any visible water boundary. The morphology of MNL observed under TEM depicted that the droplets were spherically shaped (Fig. **1E**), and the particle sizes of MNLs were about 30 nm, as shown in the TEM diagrams, wherein they were in line with the values obtained *via* DLS (33.26 ± 4.40 nm, PDI = 0.130 ± 0.017) (Fig. **1F**). Likewise, the value of the Z-potential of MNL was −5.04 ± 0.43 mV. The particle size and Z-potential of MNL showed slight changes from day 1 to day 7 with no statistical significance (*P* > 0.05) (Fig. **1G**, **H**). These findings indicated that MNL was appreciably stable. Meanwhile, the DL% and EE% were 16.14% ± 1.59 and 65.55% ± 2.47, respectively. The cumulative release of MA from the liposomal formulation in the PBS media (pH = 7.4) within 4 h was 84.79% ± 3.48, which was significantly (*P* ˂ 0.05) higher than the unencapsulated MA (45.79% ± 2.53) (Fig. **1I**). At 24 h, the respective cumulative releases of MA and MNL were 91.12% ± 2.69 and 81.02% ± 3.08.

### 3.2 MNL Inhibited Growth of KYSE-150 Cells

**Fig. (2):**
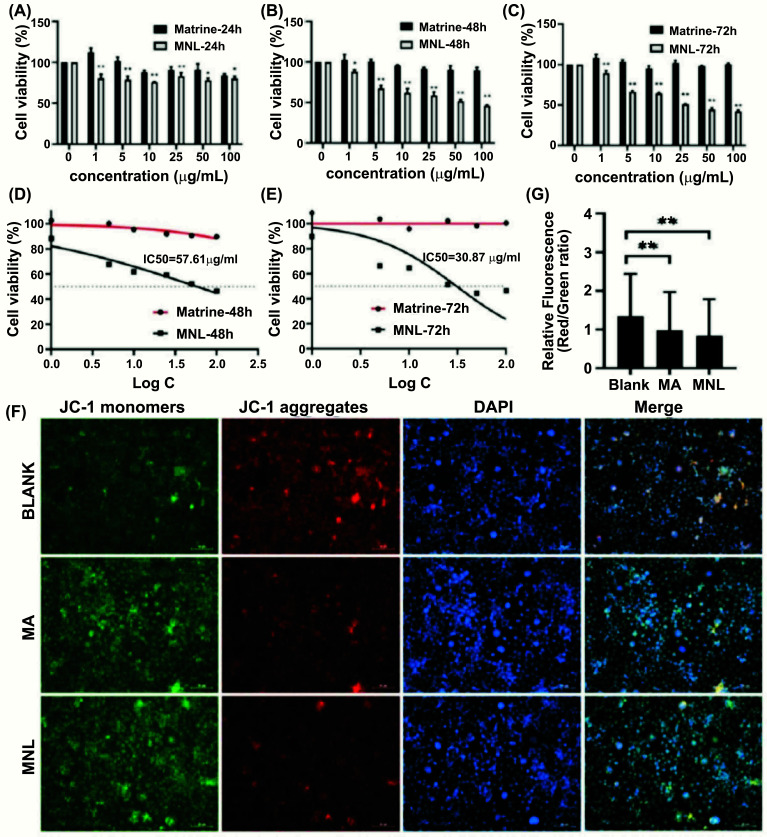
Matrine (MA)-loaded nano-liposomes (MNLs) down-regulated the cell viability of KYSE-150 and promote apoptosis. (**A**-**C**) KYSE-150 cells treated with various concentrations of MA and MNLs for 24 h, 48 h, and 72 h. Cell viability was determined using the MTT assay. (**D**, **E**) IC_50_ values were calculated from dose-response curves for MA and MNLs at 48 h and 72 h to evaluate the toxicity. (**F**, **G**) Changes in mitochondrial membrane potential were observed using fluorescence microscopy after MA and MNLs treatment. Red fluorescence indicates aggregates, while green fluorescence indicates monomers. Values are presented as mean ± standard deviation from three independent experiments. (*P < 0.05, **P < 0.01, compared to the MA or blank group).

One of the notable characteristics of cancer cells is inexhaustible growth. Hence, we designed this experiment to purposively detect the effect of MNL on the survival of KYSE-150 cells. In this experiment, an MTT assay was employed to investigate a broad range (0-100 μg/mL) of MNL concentrations. Fig. (**2A**-**C**) present a clear decreasing trend of KYSE-150 cell viability upon the treatment with MNLs at 24, 48, and 72 h. It can be observed that the cell viabilities (at 24 h) were 80.54 ± 4.93%, 78.78 ± 4.25%, 75.80 ± 0.47%, 83.21 ± 3.91%, 78.48 ± 2.16%, and 80.33 ± 2.75% after treatment with respective MNL concentrations of 1.0, 5.0, 10, 25, 50, and 100 μg/mL (Fig. **2A**-**C**). Respectively, the cell viabilities at 48 h were 88.20 ± 2.31%, 67.59 ± 3.70%, 61.92 ± 5.52%, 59.08 ± 3.80%, 52.10 ± 1.48%, and 46.29 ± 0.66%, while cell viabilities at 72 h were 89.80 ± 2.85%, 66.39 ± 1.76%, 64.56 ± 1.12%, 51.27 ± 0.21%, 44.51 ± 2.01%, and 42.19 ±1. 46% after treatment with the above-mentioned concentrations of MNLs. Noticeably, we observed marked suppression in the growth of KYSE-150 cells at prominent concentrations of MNLs (particularly 25, 50, and 100 μ*/*mL) at 48 and 72 h, wherein the viability of the cells was dose-dependently suppressed by MNLs. Meanwhile, the cell viabilities of MA treatment were all significantly higher than those of the MNL group at any time. After treating the aforementioned cells at 24 h with MNLs, we could not calculate the value of IC_50_, but their respective IC_50_ values at 48 and 72 h were approximately 57.61 and 30.87 μg/mL (Fig. **2D**, **E**). Suggestively, MNLs could substantially inhibit the proliferation of KYSE-150 cells in a dose-dependent manner at 48 and 72 h. Meanwhile, the concentration of 25 μg/mL for MNLs at 48 h was chosen for subsequent experiments.

### 3.3 MNL Induced Mitochondrial-dependent Apoptosis

Alterations in MMP were explored after treatment of the cells with MNL, wherein affirmation of the disruption was carried out using JC-1 as an indicator. Importantly, MNL induced a significant change in fluorescent color from red to green comparable to the blank batch (Fig. **2F**, **G**), suggesting that the disturbance of MMP in KYSE-150 cells was triggered by MNL. Later on, the ratio of fluorescent red to fluorescent green was calculated (Fig. **2G**). Compared to the blank group, the MA batch showed a decrease in the relative fluorescent ratio from 1.34 ± 1.10 to 0.98 ± 0.99 (*P* < 0.01), while that of the MNL treatment group also decreased from 1.34 ± 1.10 to 0.98 ± 0.99 (*P* < 0.01), with the degree of MNL decrease being the strongest. Overall, these observations suggest the potential of MNLs to induce disturbance in MMP and concomitantly promote apoptosis of the KYSE-150 cells.

### 3.4 Effect of MNL on Caspase-3, Bcl-2, and Bax Expressions

**Fig. (3):**
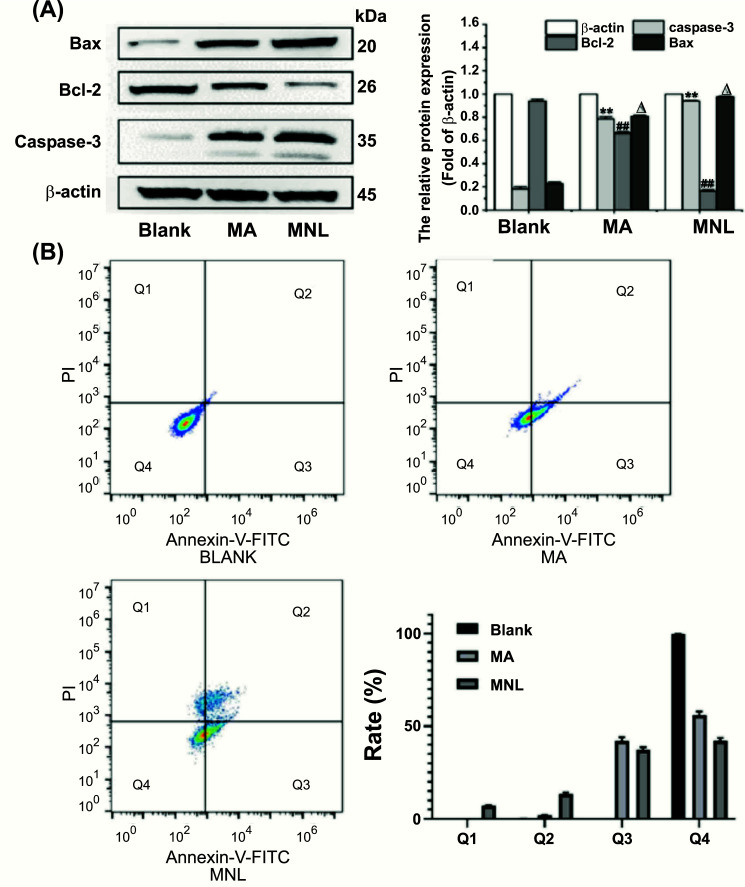
Western blot analysis and Annexin V-FITC apoptosis detection of matrine (MA) and matrine-loaded nano-liposomes (MNLs). (**A**) Western blot analysis showing the effect of MA and MNLs on the expression of Bax, Bcl-2, and caspase-3. Bax and caspase-3 expression levels increased significantly, while Bcl-2 expression decreased significantly (**P < 0.01, ##*P* < 0.01, Δ*P* < 0.01, compared to the blank group). (**B**) Apoptosis rate analysis by flow cytometry. After MA and MNLs treatments, apoptotic ratios increased significantly (**P < 0.01, compared to the blank group).

To further ascertain whether MNL promotes apoptosis in KYSE-150 cells, we further detected three proteins, namely caspase-3 (involved in permeability of mitochondria regulation), Bcl-2 (as an anti-apoptotic member), and Bax (as a pro-apoptotic member). Expressions of caspase-3, Bcl-2, and Bax were evaluated using the Western blotting technique. After treatment with MA and MNL, we observed that the expression of Bax was increased (*P* < 0.01) substantially (Fig. **3A**), whilst that of Bcl-2 was decreased markedly (*P* < 0.01). Moreover, the involvement of caspase-3 in the pathway of apoptosis was observed, indicating that the protease levels were substantially increased after treatment with MA and MNL (*P* < 0.01).

### 3.5 MNL Induced Apoptosis in KYSE-150 Cells

In recent times, various scientists have studied the anti-tumor activity of several TCMs, with a majority of the drugs inducing apoptosis in tumor cells. In this regard, we assayed MNL apoptotic rate using Annexin V-FITC/PI kit in order to understand the effect of the alkaloid on ESCC KYSE-150 cells. In terms of results (Fig. **3B**), we estimated the live cell ratio in blank batch to be 99.75% ± 0.05, whilst the proportion showed a decreasing trend (55.99% ± 1.92 and 42.11% ± 1.61, respectively) after treatment of the cells with MA and MNLs. In the meantime, we found an increase in early-stage apoptotic proportion from 0.06% ± 0.02 to 42.04% ± 1.91, 37.3% ± 1.30. Likewise, advanced apoptotic and necrotic proportion increased from 0.17% ± 0.04 to 1.95% ± 0.07 and 13.51% ± 0.85, respectively. It is obvious from the above finding that MA and MNLs could induce apoptosis in KYSE-150 cells, with MNLs enhancing the apoptotic process more than MA.

## 4 DISCUSSION

**Fig. (4):**
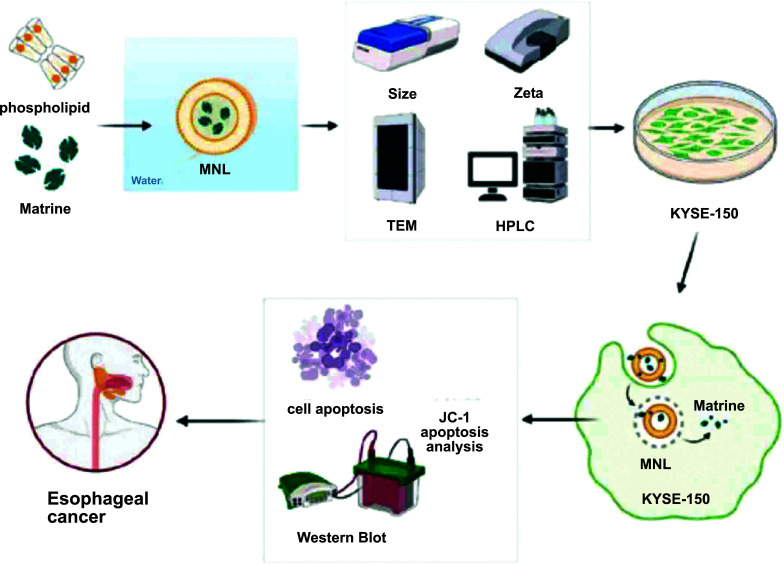
Schematic representation of the preparation and evaluation of matrine-loaded nano-liposomes (MNLs). MNLs were prepared using membrane-sonic methods and assessed for size, Z potential, transmission electron microscopy, drug loading, and encapsulation efficiency. In KYSE-150 cells, various assessments, including cell viability, JC-1 staining, Annexin V-FITC assay, and Western blot analysis, were performed. These evaluations demonstrated that MNLs promote apoptosis, suggesting that MNLs may represent a more effective anti-ESCC strategy.

Existing data suggest a wide exploration of MA, which is considered the principal active constituent of *S. tonkinensis* and *S. flavescens* Alt. roots [24]. Previous literature has identified that MA possesses several health benefits, including anti-tumor [9], anti-inflammation [25], anti-oxidation [6], neuroprotection [26], *etc*. As an anti-cancer agent, MA has been reported [13, 27-29] to demonstrate favorable anti-tumor effects, suppress metastasis and invasion, minimize radiotherapy and chemotherapy toxicity, promote apoptosis, induce cell arrest, and reverse resistance of multidrug. Hence, MA is still a worthwhile anti-tumor drug to explore. Therefore, we investigated the enhanced MA effect on anti-ESCC. As stated earlier in this work, nano-sized drug carriers help enhance the absorption of drugs and improve their therapeutic effects [22]. The reticulo-endothelial system (RES) rapidly cleared liposomes, which resulted in the high buildup of drugs at targeted sites of disease through the EPR effect. In comparison with normal tissues, the process of the EPR effect is a mechanism through which larger molecules are increasingly extravasated from the blood vessels of tumors and progressively retained in cancerous tissues [30, 31]. Therefore, we prepared MNLs with the membrane-sonic method to enhance their anti-tumor effect before applying them to KYSE-150 cells (Fig. **4**).

The aforementioned results showed that the particle size of MNLs was measured to be 33.26 ± 4.40 nm and PDI = 0.130 ± 0.017 (Fig. **2E**), wherein unimodal distribution curves of particle size were observed (Fig. **1F**). In terms of PDI, a low value was estimated, suggesting uniform sizes of nano-liposomal particles. Besides, morphological observation of MNLs using the TEM technique demonstrated that the nano-liposomal sizes were homogeneously and spherically shaped, which was consistent with the DLS results. It was reported that liposomes with small size (<50 nm diameter) could escape catabolism in the bloodstream coupled with prolonged circulation in laboratory animals and human subjects with cancer. Hence, MNLs could be regarded as passive targeting liposomes, which resist degradation in blood. Furthermore, MNLs may have better absorption at the site of tumor tissues, albeit there is a need for further research *in vivo*. The standard curve was applied to determine the concentration of MA. As an important index to evaluate nano-liposomal quality, EE (%) can have a valuable impact on the therapeutic effect of drugs. Of note, the estimated EE (%) for MNLs was 65.55% ± 2.47, which may be due to the chemical nature of MA, which is an alkaloid with good water solubility. Also, the EE (%) of small unilamellar vesicles is generally lower. During the period (7 days at 4℃) of storing MNLs, we discovered no significant changes in particle size and Z-potential (Figs. **1G**, **H**). Importantly, a slight increase in mean sizes of nano-liposomal particles (roughly within the 10 nm range) was observed. It is worth mentioning that the process of storing nano-liposomes can result in sedimentation, fusion, or agglomeration of the smaller-sized carriers, thereby affecting their mean particle sizes. Possibly, the negatively charged surface of MNLs may be due to the phospholipids present in the liposomal vesicles, which have the potential to keep the stability of nano-liposomal solution *via* electrostatic repulsion.

Under sink conditions, we evaluated the pattern of *in vitro* MA release from MNLs for 24 h at 37℃ in PBS. Fig. (**1I**) displays the result of accumulative MA release from liposomal vesicles. Within 4 h of evaluation, we discovered that free MA could release 84.79 ± 3.48% in PBS (pH 7.4). This observation may be attributable to the complete diffusion of MA throughout the dialysis membrane. In totality, roughly 91.12 ± 2.69% of MA was accumulatively and sustainably released from MNL in PBS within 24 of the evaluation. Hence, we observed an increased rate of accumulative MA release from MNLs in PBS that was comparable to free MA. Thus, this observation indicates that nano-sized particles may increase water solubility.

Furthermore, we performed the cell viability assay, JC-1 staining, Western blot assay, and apoptotic assay on KYSE-150 cells under MNL treatment. As shown in Fig. (**2A**-**C**), MNLs significantly increased the toxicity to KYSE-150 cells compared to MA at 24, 48, and 72 h, which is consistent with the trend of most studies targeting tumor cells. Contrary to our studies, a liposomal nanoparticle was found to be less toxic to tumor cells compared to a DMSO- solubilized free drug due to the toxic effects of DMSO on both malignant and non-malignant cells [32]. It is reasonable to speculate that MA-loaded nano-liposome not only reduces the toxicity of DMSO on non-malignant cells but has an enhanced toxic effect on malignant cells, reflecting the safety profile of the nano-formulation. After treating the aforementioned cells at 24 h with MNLs, we could not calculate the value of IC_50_, but its respective IC_50_ values at 48 and 72 h were approximately 57.61 and 30.87 μg/mL (Fig. **2D**, **E**). A decrease in the value of IC_50_ indicates that MNLs have higher cytotoxicity over time, while the apoptotic rate of KYSE-150 cells increased gradually. Thus, MNLs could substantially inhibit the proliferation of KYSE-150 cells in a dose-dependent manner at 48 and 72 h. Furthermore, mitochondria are associated with alteration in MMP and, therefore, are principally involved in the induction of apoptosis [33, 34]. In our study, we used JC-1 staining to affirm MMP disturbance. Comparable to MA, KYSE-150 cells that were treated with MNLs exhibited obvious apoptosis, followed by a marked decrease in MMP. Apoptosis is a programmed cell death process that can be triggered by a variety of signaling pathways, such as mitochondria-mediated pathways and the caspase cascade. For mitochondria-mediated pathways, regulation of MMP is an important process in apoptosis, with various families of proteins playing crucial roles, namely Bcl-2 (as an anti-apoptotic member) and Bax (as a pro-apoptotic member) [35]. The anti-apoptotic member Bcl-2 prevents apoptosis by controlling mitochondrial function and cytochrome c release, whereas the pro-apoptotic protein bax has the opposite effect that can promote cell death. Furthermore, it is widely accepted that the ratio between pro- and anti-apoptotic proteins is a determinant of the mitochondrial apoptotic pathway. Through this experimentation, we speculated that MA and MNLs may have inhibited KYSE-150 cells *via* dysfunction of mitochondria because the dosage forms potentially enhanced and attenuated Bax and Bcl-2 expressions, respectively. Ultimately, the morphological apoptotic hallmarks are evoked by caspase-3 by inducing fragmentation of DNA and loss of cell volume. The up-regulated level of caspase-3 also suggests that activated caspase-3 plays a key role in the apoptosis-inducing effect of MNLs on KYSE-150 cells. The results of the Annexin V-FITC/PI assay also demonstrated that MNLs promoted apoptosis in KYSE-150 cells at early and advanced stages.

## CONCLUSION

Taken together, our results indicate that MA could inhibit the growth of KYSE-150 cells and promote apoptosis. MNLs could amplify these properties because of their nano-sized structure. Thus, MNLs might represent a more effective anti-cancer strategy to enhance the anti-ESCC effects of MA. In the future, there is a need for the establishment of *in-vivo* ESCC animal models to validate the therapeutic effects of MNLs and an in-depth study on the mechanisms underlying the anti-ESCC activity of MNLs. This would provide a potentially effective natural drug and delivery form for the treatment of ESCC in the clinic.

## Figures and Tables

**Table 1:** Intra-day precision results of various concentrations of matrine (MA) (n=5).

**MA**	**Measured Concentration (μg/mL)**					**Mean**	**RSD**
**(μg/mL)**	**1**	**2**	**3**	**4**	**5**	**(μg/mL)**	**(%)**
30	29.38	29.76	30.38	30.4	29.68	29.92	1.51
100	100.01	101.04	101.89	99.34	98.48	100.15	1.35
300	302.89	301.08	302.90	300.93	299.81	301.52	0.45

**Table 2:** Inter-day precision results of various concentrations of matrine (MA) (n=5).

**MA**	**Measured Concentration (μg/mL)**					**Mean**	**RSD**
**(μg/mL)**	**1**	**2**	**3**	**4**	**5**	**(μg/mL)**	**(%)**
30	30.65	29.94	30.84	29.70	29.87	30.20	1.69
100	100.20	100.64	100.34	99.25	99.34	99.95	0.62
300	298.23	305.33	301.07	299.85	300.32	300.96	0.88

**Table 3:** Recovery test of various concentrations of matrine (MA) (n=3).

**MA**	**Recovery (%)**			**Mean **	**RSD**
**(μg/mL)**	**1**	**2**	**3**	**(μg/mL)**	**(%)**
30	99.16	100.85	98.92	99.64	1.06
100	99.31	99.92	100.12	99.78	0.42
300	99.56	100.42	99.78	99.92	0.45

**Table 4:** Results of stability of various concentrations of matrine (MA) (n=3).

**Time**	**Average Measured Values (μg/mL)**		
**(h)**	**30**	**100**	**300**
0	30.11	100.84	297.57
2	29.55	99.27	301.61
4	30.01	100.48	299.99
8	29.86	99.56	302.28
12	30.29	101.86	298.49
24	29.28	101.33	299.93
48	30.10	100.42	298.65
RSD (%)	1.19	0.91	0.57

**Table 5:** Repeatability test of various concentrations of matrine (MA) (n=5).

**MA**	**Measured Concentration (μg/mL)**					**Mean**	**RSD**
**(μg/mL)**	**1**	**2**	**3**	**4**	**5**	**(μg/mL)**	**(%)**
30	31.12	30.49	30.18	29.26	30.26	30.26	2.22
100	99.36	100.37	98.27	101.58	100.10	99.94	1.23
300	297.79	299.13	301.93	301.31	304.28	300.89	0.84

## Data Availability

All the data and supporting information are available within the article.

## LIST OF ABBREVIATIONS

EC: Esophageal Cancer
ECA: Esophageal Adenocarcinoma
FBS: Fetal-bovine Serum
HPLC: High-performance Liquid Chromatography
MNLs: MA-loaded Nano-liposomes
PAHs: Polynuclear-aromatic Hydrocarbons
PVDF: Poly-vinyl-idene Di-fluoride
RES: Reticulo-endothelial System
TCM: Traditional Chinese Medicine
TEM: Transmission Electron Microscopic

## References

[1] Pennathur A., Gibson M.K., Jobe B.A., Luketich J.D. (2013). Oesophageal carcinoma.. Lancet.

[2] Watanabe M., Otake R., Kozuki R., Toihata T., Takahashi K., Okamura A., Imamura Y. (2020). Recent progress in multidisciplinary treatment for patients with esophageal cancer.. Surg. Today.

[3] Abnet C.C., Arnold M., Wei W.Q. (2018). Epidemiology of *Esophageal squamous* cell carcinoma.. Gastroenterology.

[4] Miller K.D., Nogueira L., Mariotto A.B., Rowland J.H., Yabroff K.R., Alfano C.M., Jemal A., Kramer J.L., Siegel R.L. (2019). Cancer treatment and survivorship statistics, 2019.. CA Cancer J. Clin..

[5] Cao L., Wang X., Zhu G., Li S., Wang H., Wu J., Lu T., Li J. (2021). Traditional Chinese medicine therapy for esophageal cancer: A literature review.. Integr. Cancer Ther..

[6] Hu C., Zhang X., Wei W., Zhang N., Wu H., Ma Z., Li L., Deng W., Tang Q. (2019). Matrine attenuates oxidative stress and cardiomyocyte apoptosis in doxorubicin-induced cardiotoxicity *via* maintaining AMPK*α*/UCP2 pathway.. Acta Pharm. Sin. B.

[7] Zhaowu Z., Xiaoli W., Yangde Z., Nianfeng L. (2009). Preparation of matrine ethosome, its percutaneous permeation *in vitro* and anti-inflammatory activity *in vivo* in rats.. J. Liposome Res..

[8] Ma W., Xu J., Zhang Y., Zhang H., Zhang Z., Zhou L., Wang X., Liu H., Chen Y., Du P., Min N., Liu Z., Yin Y. (2019). Matrine pre-treatment suppresses AGEs- induced HCSMCs fibrotic responses by regulating Poldip2/mTOR pathway.. Eur. J. Pharmacol..

[9] Wang W., You R., Qin W., Hai L., Fang M., Huang G., Kang R., Li M., Qiao Y., Li J., Li A. (2015). Anti-tumor activities of active ingredients in compound kushen injection.. Acta Pharmacol. Sin..

[10] Chi G., Xu D., Zhang B., Yang F. (2019). Matrine induces apoptosis and autophagy of glioma cell line U251 by regulation of circRNA-104075/BCL-9.. Chem. Biol. Interact..

[11] Wang M., Liu G., Li H. (2018). Extraction of matrine from *Sophora flavescens Ait.* and evaluation of its inhibitory effects on human nasopharyngeal carcinoma CNE-2 cells.. Food Sci. Technol..

[12] Zhou Y., Feng J., You L., Meng H., Qian W. (2015). Matrine and CYC116 synergistically inhibit growth and induce apoptosis in multiple myeloma cells.. Chin. J. Integr. Med..

[13] Zhang J.Q., Li Y.M., Liu T., He W.T., Chen Y.T., Chen X.H., Li X., Zhou W.C., Yi J.F., Ren Z.J. (2010). Antitumor effect of matrine in human hepatoma G2 cells by inducing apoptosis and autophagy.. World J. Gastroenterol..

[14] Yang A., Zhu J., Xu F., Yang J., Wang Y., Wei M., Bai X., Yang Y. (2022). Anticancer effect of a combination of cisplatin and matrine on cervical cancer U14 cells and U14 tumor-bearing mice, and possible mechanism of action involved.. Trop. J. Pharm. Res..

[15] Jin H., Sun Y., Wang S., Cheng X. (2013). Matrine activates PTEN to induce growth inhibition and apoptosis in V600EBRAF harboring melanoma cells.. Int. J. Mol. Sci..

[16] Zhang S., Cheng B., Li H., Xu W., Zhai B., Pan S., Wang L., Liu M., Sun X. (2014). Matrine inhibits proliferation and induces apoptosis of human colon cancer LoVo cells by inactivating Akt pathway.. Mol. Biol. Rep..

[17] Li Y., Lin H., Deng N., Xie L., Luo R. (2019). Matrine from *Vietnamese sophora* root inhibits the growth of oral squamous cell carcinoma cells *in vitro* and *in vivo*.. Food Sci. Technol..

[18] Gu J., Wang X., Zhang L., Xiang J., Li J., Chen Z., Zhang Y., Chen J., Shen J. (2021). Matrine suppresses cell growth of diffuse large B-cell lymphoma *via* inhibiting CaMKIIγ/c-Myc/CDK6 signaling pathway.. BMC Complement. Med. Ther..

[19] Shao H., Yang B., Hu R., Wang Y. (2013). Matrine effectively inhibits the proliferation of breast cancer cells through a mechanism related to the NF-κB signaling pathway.. Oncol. Lett..

[20] Luo C., Zhu Y., Jiang T., Lu X., Zhang W., Jing Q., Li J., Pang L., Chen K., Qiu F., Yu X., Yang J., Huang J. (2007). Matrine induced gastric cancer MKN45 cells apoptosis *via* increasing pro-apoptotic molecules of Bcl-2 family.. Toxicology.

[21] Jiang J.H., Pi J., Jin H., Yang F., Cai J.Y. (2018). Chinese herb medicine matrine induce apoptosis in human esophageal squamous cancer KYSE-150 cells through increasing reactive oxygen species and inhibiting mitochondrial function.. Pathol. Res. Pract..

[22] Gabizon A.A., Shmeeda H., Zalipsky S. (2006). Pros and cons of the liposome platform in cancer drug targeting.. J. Liposome Res..

[23] Nikpoor A.R., Tavakkol-Afshari J., Gholizadeh Z., Sadri K., Babaei M.H., Chamani J., Badiee A., Jalali S.A., Jaafari M.R. (2015). Nanoliposome-mediated targeting of antibodies to tumors: IVIG antibodies as a model.. Int. J. Pharm..

[24] Funaya N., Haginaka J. (2012). Matrine and oxymatrine-imprinted monodisperse polymers prepared by precipitation polymerization and their applications for the selective extraction of matrine-type alkaloids from *Sophora flavescens Aiton.*. J. Chromatogr. A.

[25] Wu G., Zhou W., Zhao J., Pan X., Sun Y., Xu H., Shi P., Geng C., Gao L., Tian X. (2017). Matrine alleviates lipopolysaccharide-induced intestinal inflammation and oxidative stress *via* CCR7 signal.. Oncotarget.

[26] You L., Yang C., Du Y., Wang W., Sun M., Liu J., Ma B., Pang L., Zeng Y., Zhang Z., Dong X., Yin X., Ni J. (2020). A systematic review of the pharmacology, toxicology and pharmacokinetics of matrine.. Front. Pharmacol..

[27] Liang C.Z., Zhang J.K., Shi Z., Liu B., Shen C.Q., Tao H.M. (2012). Matrine induces caspase-dependent apoptosis in human osteosarcoma cells *in vitro* and *in vivo* through the upregulation of Bax and Fas/FasL and downregulation of Bcl-2.. Cancer Chemother. Pharmacol..

[28] Niu H., Zhang Y., Wu B., Zhang Y., Jiang H., He P. (2014). Matrine induces the apoptosis of lung cancer cells through downregulation of inhibitor of apoptosis proteins and the Akt signaling pathway.. Oncol. Rep..

[29] Li H., Li X., Bai M., Suo Y., Zhang G., Cao X. (2015). Matrine inhibited proliferation and increased apoptosis in human breast cancer MCF-7 cells *via* upregulation of Bax and downregulation of Bcl-2.. Int. J. Clin. Exp. Pathol..

[30] Maeda H., Wu J., Sawa T., Matsumura Y., Hori K. (2000). Tumor vascular permeability and the EPR effect in macromolecular therapeutics: A review.. J. Control. Release.

[31] Sawant R.R., Torchilin V.P. (2012). Challenges in development of targeted liposomal therapeutics.. AAPS J..

[32] Wang D., Veena M.S., Stevenson K., Tang C., Ho B., Suh J.D., Duarte V.M., Faull K.F., Mehta K., Srivatsan E.S., Wang M.B. (2008). Liposome-encapsulated curcumin suppresses growth of head and neck squamous cell carcinoma *in vitro* and in xenografts through the inhibition of nuclear factor kappaB by an AKT-independent pathway.. Clin. Cancer Res..

[33] Jung H., Bae J., Ko S.K., Sohn U.D. (2016). Ultrasonication processed *Panax ginseng* berry extract induces apoptosis through an intrinsic apoptosis pathway in HepG2 cells.. Arch. Pharm. Res..

[34] Wasim L., Chopra M. (2018). Synergistic anticancer effect of panobinostat and topoisomerase inhibitors through ROS generation and intrinsic apoptotic pathway induction in cervical cancer cells.. Cell Oncol..

[35] Ma Z.J., Lu L., Yang J.J., Wang X.X., Su G., Wang Z., Chen G., Sun H., Wang M., Yang Y. (2018). Lariciresinol induces apoptosis in HepG2 cells *via* mitochondrial-mediated apoptosis pathway.. Eur. J. Pharmacol..

